# Dissemination of Chest Compression-Only Cardiopulmonary Resuscitation by Bystanders for Out-of-Hospital Cardiac Arrest in Students: A Nationwide Investigation in Japan

**DOI:** 10.3390/jcm11040928

**Published:** 2022-02-10

**Authors:** Kosuke Kiyohara, Yuri Kitamura, Mamoru Ayusawa, Masahiko Nitta, Taku Iwami, Ken Nakata, Tomotaka Sobue, Tetsuhisa Kitamura

**Affiliations:** 1 Department of Food Science, Faculty of Home Economics, Otsuma Women’s University, 12 Sanbancho Chiyoda-ku, Tokyo 102-8357, Japan; 2 Division of Environmental Medicine and Population Sciences, Department of Social and Environmental Medicine, Graduate School of Medicine, Osaka University, 2-2 Yamadaoka, Suita 565-0871, Japan; ytkitamura@envi.med.osaka-u.ac.jp (Y.K.); tsobue@envi.med.osaka-u.ac.jp (T.S.); lucky_unatan@yahoo.co.jp (T.K.); 3 Department of Pediatrics and Child Health, Nihon University School of Medicine, 30-1 Oyaguchi-kamicho, Itabashi-ku, Tokyo 173-8610, Japan; ayusawa.mamoru@nihon-u.ac.jp; 4 Department of Emergency Medicine, Osaka Medical College, 2-7 Daigakumachi, Takatsuki 569-8686, Japan; nittam@osaka-med.ac.jp; 5 Department of Pediatrics, Osaka Medical College, 2-7 Daigakumachi, Takatsuki 569-8686, Japan; 6 Kyoto University Health Service, Yoshida-Honmachi, Sakyo-ku, Kyoto 606-8501, Japan; iwami.taku.8w@kyoto-u.ac.jp; 7 Medicine for Sports and Performing Arts, Department of Health and Sport Sciences, Graduate School of Medicine, Osaka University, 2-2 Yamadaoka, Suita 565-0871, Japan; ken.nakata7@gmail.com

**Keywords:** out-of-hospital cardiac arrest, school, students, chest compression-only cardiopulmonary resuscitation, cardiopulmonary resuscitation with rescue breathing

## Abstract

We aimed to investigate how the types of bystander-initiated cardiopulmonary resuscitation (CPR) for out-of-hospital cardiac arrest (OHCA) among students have changed recently. We also determined the association between two types of bystander-CPRs (i.e., chest compression-only CPR [CCCPR] and conventional CPR with rescue breathing [CCRB]) and survival after OHCA. From a nationwide registry of pediatric OHCAs occurring in school settings in Japan, the data of 253 non-traumatic OHCA patients (elementary, junior high, and high school/technical college students) receiving bystander-CPR between April 2008 and December 2017 were analyzed. Multivariable logistic regression analysis was conducted to assess the impact of different types of bystander-CPR on 30-day survival with favorable neurological outcomes. The proportion of patients receiving CCCPR increased from 25.0% during 2008–2009 to 55.3% during 2016–2017 (*p* for trend < 0.001). Overall, 53.2% (50/94) of patients receiving CCCPR and 46.5% (74/159) of those receiving CCRB survived for 30 days with favorable neurological outcomes. Multivariable analysis showed no significant difference in outcomes between the two groups (adjusted odds ratio: 1.23, 95% confidence interval: 0.67–2.28). In this setting, CCCPR is a common type of bystander-CPR for OHCA in students, and the effectiveness of CCCPR and CCRB on survival outcomes seems comparable.

## 1. Introduction

The occurrence of out-of-hospital cardiac arrest (OHCA) in school-aged children is a tragic event with a strong social impact [1]. Therefore, it is crucial for bystanders to recognize OHCA immediately, initiate cardiopulmonary resuscitation (CPR), and use an automated external defibrillator (AED) as early as possible to successfully link “The Chain of Survival” [2].

The concept of chest compression-only CPR (CCCPR) has emerged as an alternative to conventional CPR with rescue breathing (CCRB). Because CCCPR is simpler and therefore easy to teach and implement [3,4], it has the potential to improve the rate of CPR implementation and overall survival of the patient. Although both types of bystander-CPR are undoubtedly lifesaving in pediatric OHCA patients, previous studies have suggested that CCCPR appears to be as effective as CCRB for adults with OHCA [5,6,7,8,9,10,11,12,13], whereas CCCPR may be less effective for children whose OHCAs most commonly result from respiratory causes [14,15,16,17]. Therefore, the latest Japanese CPR guidelines recommend CCCPR for untrained bystanders and those unwilling to perform rescue breaths for adults with OHCA but recommend CCRB for pediatric OHCA instead of CCCPR.

However, it is still debated whether CCCPR or CCRB is more appropriate as an initial intervention for pediatric OHCA patients by bystanders. In the past decade, the benefits of CCCPR and CCRB for pediatric OHCA patients have been examined in several observational studies [14,15,16,18], but these studies did not include information about specific locations of arrest. Therefore, the current status of implementation of bystander-CPR and its effects have not been adequately investigated in school settings. Because school-age children spend a large part of their active daytime at school or in other public places [19], it is important to accumulate scientific data from school settings.

The Japanese Circulation Society has proposed “Aiming for zero death” to achieve the goal of zero sudden cardiac deaths in schools [20]. However, OHCA among students has been shown to have a higher survival rate than in OHCA in other locations, but it is still far from being lifesaving for all patients [21]. In Japan, at least one AED is installed in most schools; more than 90% of schools provide CPR training for teachers and staff and promote CPR education for students [22]. Furthermore, most OHCAs among students are being witnessed by bystanders, are of cardiac origin, and with a ventricular fibrillation (VF) rhythm [23]. Thus, pediatric OHCAs occurring in school settings differ greatly from those occurring in other locations, in terms of the surrounding environment and patient characteristics. Therefore, understanding the actual situation of bystander-CPR and the effect of type of CPR on survival outcomes in schools is useful in establishing CPR education programs that are appropriate for this setting.

Stop and Prevent cardIac aRrest, Injury, and Trauma in Schools (SPIRITS) is a study that constructs and analyzes a nationwide registry of pediatric OHCAs occurring in school settings in Japan [23,24,25]. In the present study, we aimed to (1) clarify how the types of bystander-initiated CPR (i.e., CCCPR or CCRB) for OHCA among students have changed in recent years, and (2) to investigate the association between the two types of bystander-CPR and survival after OHCA using data from the SPIRITS registry.

## 2. Materials and Methods

### 2.1. Study Design of SPIRITS

The study profiles of SPIRITS have been previously described in detail [23]. Briefly, SPIRITS constructs and analyzes an OHCA registry that includes data collected from two nationwide databases: the Injury and Accident Mutual Aid Benefit System of the Japan Sport Council, and the All-Japan Utstein Registry of the Fire and Disaster Management Agency. Individual patient data from each database were combined into a single database. Data collection for the SPIRITS began in April 2008. The Injury and Accident Mutual Aid Benefit System provides medical expenses, disability compensation, and death compensation for injuries, illnesses, diseases, accidents, and deaths that occur in children and students attending schools. It covers most children and students attending schools in Japan (82.0% of certified nursery school pupils/kindergarteners, 99.9% of elementary school students, 99.9% of junior high school students, and 97.6% of high school and technical college students in 2017, covering approximately 16.7 million children and students) [26]. Data on approximately one million injuries and accidents were registered annually from 55,500 schools [26]. The All-Japan Utstein Registry is a nationwide registry of OHCA for the entire Japanese population based on the International Utstein format [27,28]. In the registry, OHCA data are recorded by emergency medical service (EMS) personnel, in collaboration with the patient’s physician in charge. Cardiac arrest was defined as the cessation of cardiac mechanical activity, as confirmed by the absence of signs of circulation. Because prehospital termination of resuscitation by EMS personnel is not generally allowed, most OHCA patients treated by EMS personnel are transported to hospitals and the data are recorded in the registry, with the exception of OHCA patients who were not transported to the hospital by EMS. Thus, the SPIRITS registry, which was created by combining the two databases described above, contains data on most pediatric OHCAs occurring in school settings in Japan.

### 2.2. Study Subjects

This study enrolled patients with OHCA that occurred in elementary school (age 6–12 years), junior high school (age 12–15 years), high school (age ≥ 15 years), and technical college (age ≥ 15 years) students in Japan between 1 April 2008 and 13 December 2017. Patients for whom CPR was attempted by bystanders and EMS personnel and the first documented rhythm was recorded were included. Cases of OHCA occurring after the arrival of EMS personnel, those occurring outside the school campus, and those occurring due to traumatic causes (traffic accidents, falling incidents, and hanging) were excluded from the analyses. The protocol was approved by the Ethics Committee of Otsuma Women’s University (02-022). Personal identifiers were removed beforehand from the database, and the requirement for individual informed consent was waived.

### 2.3. Data Collection and Outcome Measures

Information of the following variables was obtained from the SPIRITS registry: sex, age, date of occurrence, cause of arrest, location of arrest, witness of OHCA, first documented rhythm assessed by EMS personnel after arrival at the scene, initiation of bystander-CPR, application of public-access AED pads, dispatcher instruction, and survival outcomes after OHCA. The primary outcome of the present study was 30-day survival after the occurrence of OHCA with a favorable neurological outcome. All survivors of OHCA were followed up for up to 30-days, and the neurological status was scored by the physician in charge, using the Glasgow–Pittsburgh cerebral performance category (CPC) scale: category 1, good performance; category 2, moderate disability; category 3, severe cerebral disability; category 4, coma/vegetative state; and category 5, death/brain death. Here, 30-day survival with favorable neurological outcome was defined as CPC 1 or 2 [27,28]. Secondary outcomes were return of spontaneous circulation before arrival at the hospital and 30-day survival after OHCA.

### 2.4. Statistical Analysis

Eligible OHCA cases were divided into two groups according to the type of bystander-CPR they received (i.e., CCCPR or CCRB). The yearly changes in the type of bystander-CPR performed on OHCA patients during the study period were assessed using the Mantel-Haenszel χ2 test of linear association. Differences in patient characteristics and outcomes between the groups were assessed using the χ2 test. Univariable and multivariable logistic regression analyses were also conducted to assess the impact of the type of bystander-CPR on 30-day survival with favorable neurological outcome. In the multivariate analysis, odds ratios (ORs) and their associated 95% confidence intervals (CIs) were calculated, after adjusting for potential confounding factors including sex (male or female), education level (elementary school, junior high school, or high school/technical college), first documented rhythm (VF or non-VF), origin of arrest (cardiac or non-cardiac origin), witness of arrest (witnessed by bystanders or not witnessed), activity at the time of arrest (health and physical education class, other classes, athletic club activity, or other situations), location of arrest (schoolyard, gymnasium/pool, classroom, or other locations), AED pad application by bystander (yes or no), and dispatcher instruction (yes or no). Furthermore, in a subgroup analysis, the subjects were stratified by important factors of survival (first documented rhythm, origin of arrest, witness of arrest, and education level), and the association between type of bystander-CPR and survival outcomes was assessed using the χ^2^ test. All tests were two-tailed, and a *p*-value of < 0.05 was considered statistically significant. All statistical analyses were conducted using SPSS v26.0 J (IBM Corp., Armonk, NY, USA).

## 3. Results

**Figure 1:**
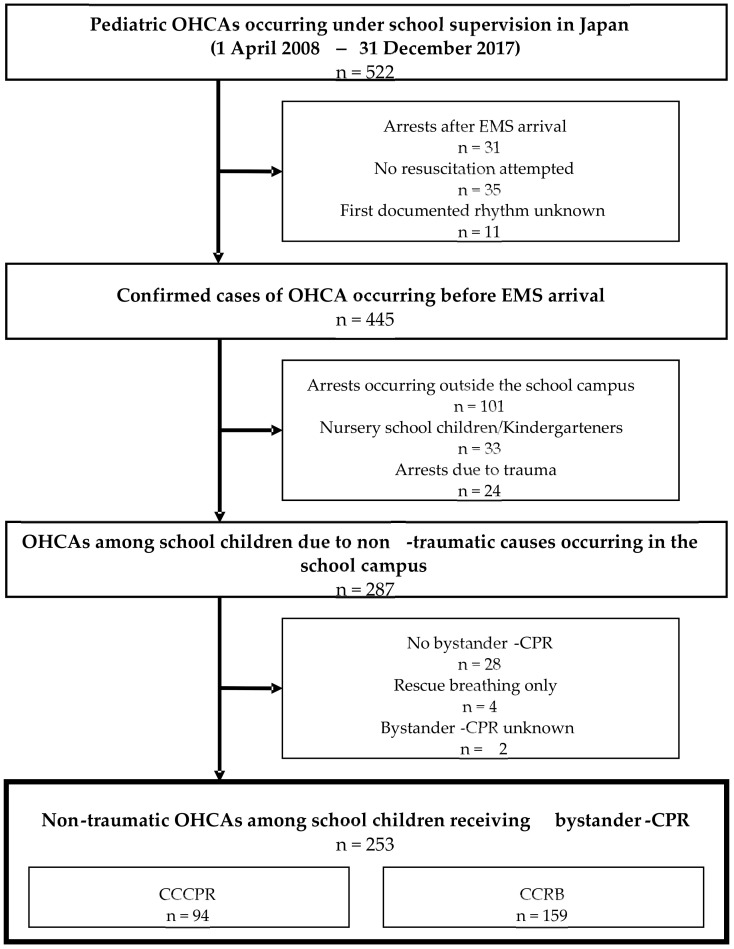
Study flowchart of the selection of pediatric out-of-hospital cardiac arrest (OHCA) cases occurring in the school campus in Japan between 1 April 2008, and 31 December 2017. EMS, emergency medical service; CCCPR, chest compression-only cardiopulmonary resuscitation; CPR, cardiopulmonary resuscitation; CCRB, conventional cardiopulmonary resuscitation with rescue breathing.

Figure 1 shows a flowchart of the selection of eligible OHCA patients in this study. A total of 287 cases of non-traumatic OHCA in students occurring on the school campus were identified during the study period between April 2008 and December 2017. Among them, 253 (88.2%) received bystander-CPR (94 received CCCPR and 159 received CCRB).

**Figure 2:**
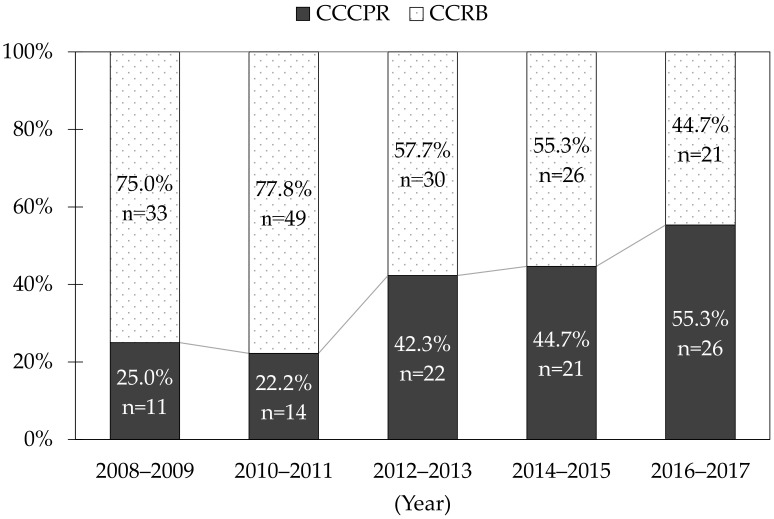
Trends in the proportion of OHCA patients who received CCCPR and CCRB during the study period. CCCPR, chest compression-only cardiopulmonary resuscitation; CCRB, conventional cardiopulmonary resuscitation with rescue breathing; OHCA, out-of-hospital cardiac arrest.

Figure 2 shows the trends in the proportion of OHCA patients receiving CCCPR and CCRB during the study period. The proportion of patients who received CCCPR significantly increased throughout the study period, from 25.0% during 2008–2009 to 55.3% during 2016–2017 (*p* for trend < 0.001).

Table 1 shows the patient characteristics and survival outcomes according to the type of bystander-CPR. In total, the majority of OHCAs had VF rhythm (82.6%), were of cardiac origin (90.1%), were witnessed by a bystander (89.3%), and involved AED pad application by bystanders (85.8%). Approximately half of the patients survived for 30 days with a favorable neurological outcome (49.0%). All characteristics and outcomes considered in this study were similar across the types of bystander-CPR performed on patients. The proportion of patients with a 30-day survival with a favorable neurologic outcome after OHCA was 53.2% in patients receiving CCCPR and 46.5% in those receiving CCRB.

Table 2 shows the factors associated with 30-day survival and a favorable neurological outcome after OHCA. In the multivariable analysis, there was no significant difference in the outcome between CCCPR and CCRB (adjusted OR: 1.23; 95% CI: 0.67–2.28). Among the variables included in the analysis, VF as the first documented rhythm (adjusted OR: 10.67, 95% CI: 2.57–44.33) and AED pad application by bystanders (adjusted OR: 3.32, 95% CI: 1.24–8.86) were the significant prognostic factors.

Table 3 shows the association between the type of bystander-CPR and survival outcomes according to first documented rhythm, origin of arrest, witness of arrest, and education level. There was no significant difference in 30-day survival with a favorable neurologic outcome between CCCPR and CCRB in any of the subgroups.

## 4. Discussion

Using data from the SPIRITS registry, the present study examined trends in the type of CPR performed by bystanders and its impact on survival outcomes among pediatric patients with non-traumatic OHCA occurring in schools during 2008–2017. We found that CCCPR is increasingly being performed by bystanders for OHCA in students, and CCCPR has no significant difference in effectiveness to CCRB. Although the incidence of OHCA in this age group is low [1,15,29], our data accumulation system, which covers most schools in Japan, enabled us to perform a national-level epidemiological study in this setting. To the best of our knowledge, this is the first study to assess the effectiveness of CCCPR and CCRB, focusing on pediatric OHCAs occurring in schools. The results of this study should have important implications for bystander CPR education and training in the future.

In this study, CCCPR showed no significant difference in 30-day survival with a favorable neurological outcome to CCRB. Recently, Naim et al., analyzed non-traumatic pediatric OHCA cases (patients aged ≤18 years) in the United States and reported that CCRB was associated with better outcomes than CCCPR in the overall pediatric cohort, but there was no association in adolescents aged ≥12 years [14]. From a nationwide OHCA registry in Japan, Kitamura et al., revealed that CCRB produced more favorable neurological outcomes than CCCPR among OHCA patients aged 1–17 years who had arrests of non-cardiac origin, but CCCPR and CCRB had similar outcomes in patients experiencing arrests of cardiac origin [15]. Similarly, Goto et al., examined pediatric OHCA patients aged <18 years in Japan and suggested that CCRB was associated with better outcomes than CCCPR; however, there was no difference between CCCPR and CCRB in OHCA of cardiac origin, VF rhythm, or age >8 years [16]. Thus, the superiority of CCCPR and CCRB would depend on patient characteristics, such as age, origin of arrest, and first documented rhythm. Importantly, this study demonstrated that most of the non-traumatic OHCA cases among students were of cardiac origin and involved a VF rhythm. Therefore, in most cases, CCCPR may be as effective as CCRB in school settings. These data would be important to dispel the concern that dissemination of CCCPR might decrease the chance of survival for victims, among whom CCRB would be more effective than CCCPR.

Our results showed that the proportion of OHCA patients who received CCCPR by bystanders significantly increased during the study period. Even though the current CPR guidelines recommend CCRB for pediatric OHCA instead of CCCPR [30], this increase in CCCPR in pediatric OHCA was also observed in reports from the United States [14] and Japan [16]. Although the reasons for the changes in the type of bystander-CPR over time are probably multifactorial, it could be partially explained by the changes in the implementation of CPR training to the general public. During the study period, the Japanese CPR guidelines started to recommend CCCPR training for the general public to further disseminate CPR by bystanders [31], based on a consensus that any CPR would be better than no CPR. In addition, Nord et al., reported that CCCPR was performed in an equal manner among both lay bystanders and bystanders with medical education, implying that even highly trained bystanders often perform CCCPR even if they are trained to provide rescue breaths [32]. Thus, CCCPR may have been widely disseminated even in school settings in Japan, where many teachers and staff are trained in basic life support [22].

During the entire study period, the proportion of pediatric OHCA patients who received bystander-CPR in schools was 88.2%, which was much higher than that in all locations in Japan [21]. However, there is still room for improvement of bystander actions, not only increasing the proportion of bystander-CPR but also shortening the time to initiation of CPR or minimizing the interruption of chest compressions to fulfill the stated aim of the AED Committee of the Japanese Circulation Society: “zero sudden cardiac deaths in schools” [20]. To improve the survival rate, further analyses are required to identify the factors of initiation of bystander-CPR and AED pad application. Importantly, as shown in our previous report, the proportion of CPR performed by bystanders in school has not changed over the past 10 years [25]. In Japan, more than 90% of schools provided CPR training for teachers and staff in 2015, while the proportion of schools providing CPR training for all students was low, at approximately 4% in elementary schools and less than 30% in junior high and high schools [22]. Because students may be the first responders to an OHCA occurring in schools, it is also necessary to provide CPR education to them. Considering that CCCPR is simpler and easier to learn and perform [3], it would be suitable for conducting CPR training programs for students. However, it should be noted that our results did not deny the additional effect of rescue breathing on survival from OHCA of respiratory origin among children as a number of previous studies have shown [14,15,16,17]. Although there were few patients with pediatric OHCA of non-cardiac origin who benefitted from CCRB in this study, some OHCA patients in a school setting would need rescue breathing. Therefore, teachers and staffs should be encouraged to learn CCRB.

This study has some inherent limitations. First, unmeasured confounding factors may have influenced the association between the type of bystander-CPR and outcomes after OHCA. This information includes past medical history, current medications, in-hospital care, quality of bystander-CPR, characteristics of bystanders (e.g., age, affiliations, sex, and experience with previous CPR training), and other relevant lifestyle habits. Second, a definitive diagnosis was not made for all cases in this study. Many cases were probably of cardiac origin, after excluding other possibilities in the All-Japan Utstein Registry. Autopsy was not performed for all cases of sudden cardiac death, and the reported autopsy rate in 2014 was only 2.4% of all deaths in Japan [33]. Therefore, in this study, several investigators, including emergency physicians and pediatricians, determined the cause of arrest by analyzing the information of diagnoses from the Injury and Accident Mutual Aid Benefit System and the All-Japan Utstein Registry [23]. Hence, there is still a possibility that unmeasured inherited functional or structural cardiac diseases and cardiac electrical disorders existed as the underlying cause of cardiac arrest, similar to those reported in previous studies [34,35,36,37]. Third, as stated in a previous paper [23], the SPIRITS registry may have been underreporting OHCA cases to a certain degree. This is due to possible input errors in the data linkage items for the development of the SPIRITS registry and the fact that cases that were not transported to the hospital by EMS were unregistered. Fourth, because of the small number of study subjects in the subgroup analysis, multivariable analysis could not be performed to assess the impact of different types of bystander-CPR. The American Heart Association Guidelines for Cardiopulmonary Resuscitation and Emergency Cardiovascular Care state that pediatric life support guidelines apply to all children between the ages of 0 and 18, but also recommend that adult basic life support guidelines should be followed for those with signs of puberty and beyond [38]. Therefore, it is especially important to analyze OHCA patients according to their education level. Prospective registration of OHCA cases in the SPIRITS database and comprehensive investigations on this topic is a future research issue.

## 5. Conclusions

In recent years, CCCPR has been increasingly performed by bystanders for OHCA in students in Japan. Considering that the majority of non-traumatic OHCAs among students were of cardiac origin and had a VF rhythm, and that the outcomes after OHCA were similar between patients receiving CCCPR and CCRB, it is reasonable to disseminate CCCPR, which is relatively easy to learn and practice in this setting.

## Figures and Tables

**Table 1:** Patient characteristics and outcomes of non-traumatic OHCA among students according to type of bystander-CPR.

Characteristics	Total		Type of Bystander-CPR				*p*-Value
			CCCPR		CCRB		
	n = 253		n = 94		n = 159		
Patient characteristics							
Male	197	(77.9%)	79	(84.0%)	118	(74.2%)	0.069
Education level							0.050
Elementary school (age, years: median 10, range 6–12)	43	(17.0%)	9	(9.6%)	34	(21.4%)	
Junior high school (age, years: median 14, range 12–15)	80	(31.6%)	31	(33.0%)	49	(30.8%)	
High school/technical college (age, years: median 17, range 15–19)	130	(51.4%)	54	(57.4%)	76	(47.8%)	
VF as first documented rhythm	209	(82.6%)	80	(85.1%)	129	(81.1%)	0.420
Cardiac origin	228	(90.1%)	88	(93.6%)	140	(88.1%)	0.152
Arrest witnessed by bystanders	226	(89.3%)	85	(90.4%)	141	(88.7%)	0.664
Situation at the time of arrest							0.892
Health and physical education class	108	(42.7%)	41	(43.6%)	67	(42.1%)	
Other classes	25	(9.9%)	8	(8.5%)	17	(10.7%)	
Athletic club activity	90	(35.6%)	35	(37.2%)	55	(34.6%)	
Other situations	30	(11.9%)	10	(10.6%)	20	(12.6%)	
Location of arrest							0.583
School yard	139	(54.9%)	48	(51.1%)	91	(57.2%)	
Gymnasium/Pool	77	(30.4%)	31	(33.0%)	46	(28.9%)	
Classroom	21	(8.3%)	10	(10.6%)	11	(6.9%)	
Other locations	16	(6.3%)	5	(5.3%)	11	(6.9%)	
AED pad application by bystanders	217	(85.8%)	82	(87.2%)	135	(84.9%)	0.608
Dispatcher instruction	121	(47.8%)	48	(51.1%)	73	(45.9%)	0.428
Survival outcomes							
Prehospital ROSC	126	(49.8%)	53	(56.4%)	73	(45.9%)	0.107
30-day survival	146	(57.7%)	57	(60.6%)	89	(56.0%)	0.468
CPC 1 or 2	124	(49.0%)	50	(53.2%)	74	(46.5%)	0.307

CCCPR, chest compression-only cardiopulmonary resuscitation; CPR, cardiopulmonary resuscitation; CCRB, conventional cardiopulmonary resuscitation with rescue breathing; AED, automated external defibrillator; CPC, cerebral performance category; VF, ventricular fibrillation; OHCA, out-of-hospital cardiac arrest.

**Table 2:** Factors associated with 30-day survival with favorable neurological outcome after non-traumatic OHCA among students.

Factors	CPC 1 or 2		Univariable Analysis		Multivariable Analysis	
	n/N	(%)	Crude OR	(95% CI)	Adjusted OR	(95% CI)
Type of bystander-CPR						
CCCPR	50/94	(53.2%)	1.31	(0.78–2.18)	1.23	(0.67–2.28)
CCRB	74/159	(46.5%)	ref.		ref.	
Sex						
Male	103/197	(52.3%)	1.83	(0.99–3.36)	0.96	(0.41–2.25)
Female	21/56	(37.5%)	ref.		ref.	
Education level						
Elementary school	12/43	(27.9%)	0.38	(0.18–0.80)	1.08	(0.33–3.46)
Junior high school	46/80	(57.5%)	1.31	(0.75–2.30)	1.26	(0.66–2.42)
High school/technical college	66/130	(50.8%)	ref.		ref.	
First documented rhythm						
VF	121/209	(57.9%)	18.79	(5.64–62.64)	10.67	(2.57–44.33)
Non-VF	3/44	(6.8%)	ref.		ref.	
Origin of arrest						
Cardiac origin	121/228	(53.1%)	8.29	(2.41–28.49)	1.19	(0.20–7.27)
Non-cardiac origin	3/25	(12.0%)	ref.		ref.	
Witness of arrest						
Witnessed by bystanders	115/226	(50.9%)	2.07	(0.89–4.81)	1.61	(0.51–5.10)
Not witnessed	9/27	(33.3%)	ref.		ref.	
Situation at the time of arrest						
Health and physical education class	70/108	(64.8%)	ref.		ref.	
Other classes	3/25	(12.0%)	0.07	(0.02–0.26)	0.21	(0.04–1.03)
Athletic club activity	46/90	(51.1%)	0.57	(0.32–1.01)	0.48	(0.25–0.93)
Other situations	5/30	(16.7%)	0.11	(0.04–0.31)	0.33	(0.09–1.19)
Location of arrest						
School yard	73/139	(52.5%)	ref.		ref.	
Gymnasium/pool	48/77	(62.3%)	1.50	(0.85–2.64)	1.70	(0.86–3.33)
Classroom	1/21	(4.8%)	0.05	(0.01–0.35)	0.22	(0.02–2.36)
Other locations	2/16	(12.5%)	0.13	(0.03–0.59)	0.32	(0.05–1.93)
AED pad application by bystanders						
Yes	116/217	(53.5%)	4.02	(1.75–9.22)	3.32	(1.24–8.86)
No	8/36	(22.2%)	ref.		ref.	
Dispatcher instruction						
Yes	62/121	(51.2%)	1.19	(0.72–1.94)	1.38	(0.76–2.50)
No	62/132	(47.0%)	ref.		ref.	

CCCPR, chest compression-only cardiopulmonary resuscitation; CPR, cardiopulmonary resuscitation; CCRB, conventional cardiopulmonary resuscitation with rescue breathing; AED, automated external defibrillator; CPC, cerebral performance category; VF, ventricular fibrillation; OHCA, out-of-hospital cardiac arrest.

**Table 3:** Association between type of bystander-CPR and survival outcomes according to first documented rhythm, origin of arrest, witness of arrest, and education level.

		Type of Bystander-CPR	Prehospital ROSC			30-Day Survival			CPC 1 or 2		
			n/N	(%)	*p*-Value	n/N	(%)	*p*-Value	n/N	(%)	*p*-Value
First documented rhythm	VF	CCCPR	53/80	(66.3%)	0.054	57/80	(71.3%)	0.140	50/80	(62.5%)	0.288
		CCRB	68/129	(52.7%)		79/129	(61.2%)		71/129	(55.0%)	
	Non-VF	CCCPR	0/14	(0.0%)	0.105	0/14	(0.0%)	0.014	0/14	(0.0%)	0.220
		CCRB	5/30	(16.7%)		10/30	(33.3%)		3/30	(10.0%)	
Origin Of arrest	Cardiac origin	CCCPR	51/88	(58.0%)	0.202	56/88	(63.6%)	0.386	49/88	(55.7%)	0.531
		CCRB	69/140	(49.3%)		81/140	(57.9%)		72/140	(51.4%)	
	Non-cardiac origin	CCCPR	2/6	(33.3%)	0.539	1/6	(16.7%)	0.258	1/6	(16.7%)	0.687
		CCRB	4/19	(21.1%)		8/19	(42.1%)		2/19	(10.5%)	
Witness of arrest	Witnessed by bystanders	CCCPR	52/85	(61.2%)	0.028	54/85	(63.5%)	0.314	48/85	(56.5%)	0.192
		CCRB	65/141	(46.1%)		80/141	(56.7%)		67/141	(47.5%)	
	Not witnessed	CCCPR	1/9	(11.1%)	0.083	3/9	(33.3%)	0.411	2/9	(22.2%)	0.386
		CCRB	8/18	(44.4%)		9/18	(50.0%)		7/18	(38.9%)	
Education level	Elementary school	CCCPR	5/9	(55.6%)	0.269	5/9	(55.6%)	0.440	4/9	(44.4%)	0.214
		CCRB	12/34	(35.3%)		14/34	(41.2%)		8/34	(23.5%)	
	Junior high school	CCCPR	18/31	(58.1%)	0.428	19/31	(61.3%)	0.580	17/31	(54.8%)	0.702
		CCRB	24/49	(49.0%)		33/49	(67.3%)		29/49	(59.2%)	
	High school/ Technical college	CCCPR	30/54	(55.6%)	0.440	33/54	(61.1%)	0.506	29/54	(53.7%)	0.573
		CCRB	37/76	(48.7%)		42/76	(55.3%)		37/76	(48.7%)	

ROSC, return of spontaneous circulation; CCCPR, chest compression-only cardiopulmonary resuscitation; CPR, cardiopulmonary resuscitation; CCRB, conventional cardiopulmonary resuscitation with rescue breathing; CPC, cerebral performance category; VF, ventricular fibrillation.

## Data Availability

Not applicable.
